# The Relationship Between Social Anxiety Disorder and ADHD in Adolescents and Adults: A Systematic Review

**DOI:** 10.1177/10870547241247448

**Published:** 2024-04-23

**Authors:** Siri Jakobsson Støre, Nejra Van Zalk, Wilma Granander Schwartz, Victoria Nilsson, Maria Tillfors

**Affiliations:** 1 Karlstad University, Sweden; 2 Child and Adolescent Psychiatry, Region Värmland, Karlstad, Sweden; 3 Imperial College London, UK; 4 Psychological Clinic for Parent and Child Health Care, Region Värmland, Karlstad, Sweden; 5 Pediatric and Adolescent Medicine, Region Värmland, Karlstad, Sweden

**Keywords:** ADHD, comorbidity, SAD, subgroup, systematic review

## Abstract

**Objective:**

This review aimed to systematically gather empirical data on the link between social anxiety disorder and ADHD in both clinical and non-clinical populations among adolescents and adults.

**Method:**

Literature searches were conducted in PsycInfo, PubMed, Scopus, and Web of Science, resulting in 1,739 articles. After screening, 41 articles were included. Results were summarized using a narrative approach.

**Results:**

The prevalence of ADHD in adolescents and adults with SAD ranged from 1.1% to 72.3%, while the prevalence of SAD in those with ADHD ranged from 0.04% to 49.5%. Studies indicate that individuals with both SAD and ADHD exhibit greater impairments. All studies were judged to be of weak quality, except for two studies which were rated moderate quality.

**Discussion:**

Individuals with SAD should be screened for ADHD and vice versa, to identify this common comorbidity earlier. Further research is needed to better understand the prevalence of comorbid ADHD and SAD in adolescents.

## Introduction

ADHD is a neurodevelopmental disorder of which the core symptoms are inattention, impulsivity, and hyperactivity. In addition to the core symptoms, there are commonly associated deficits in executive functions, such as working memory, planning, cognitive flexibility, as well as emotional regulation and social skills (American Psychiatric Association [APA], 2013; Halldorsdottir & Ollendick, 2014; Hinshaw, 2003). There are three different presentations of ADHD. The most common form is called the combined presentation, which includes symptoms of both inattention and hyperactivity/impulsivity. Additionally, there is a predominantly inattentive presentation and a predominantly hyperactive-impulsive presentation. To receive a diagnosis of ADHD, the symptoms must have been present in childhood and persist over time and across different settings. Furthermore, the symptoms must significantly impair the person’s daily functioning (APA, 2013).

Social anxiety disorder (SAD), or social phobia, is characterized by a fear of being judged by others (APA, 1994, 2013). Social situations are perceived as so intimidating that they are endured with intense anxiety or entirely avoided, even though the affected person rationally knows that the fear is exaggerated compared to the actual danger of the social situation. To receive a diagnosis of social anxiety, the fear, anxiety, or avoidance must cause clinically significant distress or impairments in important areas of life, such as relationships, work, or school. Additionally, the symptoms should have persisted for at least 6 months and should not be better explained by any other disorder (APA, 2013). The severity of social anxiety, both on diagnostic and sub-diagnostic levels, can vary from specific social situations to encompassing various types of performance and interaction situations (e.g., Spence & Rapee, 2016). According to DSM-5 (APA, 2013), as opposed to previous DSM editions, a clinical diagnosis should specify whether the fear is limited to performing in public (i.e., a performance only subgroup), instead of specifying whether the fear is present in most social situations (i.e., a generalized subgroup; APA, 1987, 1994). The more generalized form of social anxiety often leads to greater functional impairments in daily life, earlier onset of symptoms, and higher comorbidity with other disorders (Burstein et al., 2011). Hence, social anxiety can be said to exist on a continuum of severity ranging from symptoms of social anxiety up to clinical levels (i.e., SAD). This in turn indicates the importance of examining social anxiety both on diagnostic and sub-diagnostic levels.

The clinical presentation of social anxiety typically involves characteristics such as shyness, inhibition, withdrawal, and cautious behavior (Spence & Rapee, 2016). However, research has discovered a specific subgroup of people with social anxiety who do not fit this usual pattern of reserved behavior (Kachin & Pincus, 2001; Kashdan & Hofmann, 2008; Lipton et al., 2016). Instead, these individuals display impulsive actions, which can appear as assertiveness, aggressiveness, or a willingness to take risks. This unique form of social anxiety is often described as a type of social anxiety characterized by anxiety-driven impulsivity (Tillfors et al., 2021). This subgroup has been observed in adults seeking clinical help (Kachin et al., 2001), as well as in general populations of adolescents (Tillfors et al., 2013; Van Zalk et al., 2020). Approximately one in five persons with social anxiety falls into this highly impulsive subgroup of social anxiety (Kashdan & McKnight, 2010). Nonetheless, there are still many gaps in understanding of this subgroup, and further research is required (Tillfors et al., 2021).

ADHD and SAD are two of the most common psychiatric disorders today (Kessler et al., 2006). Even though they are commonly thought of as widely different, with shy traits often contrasted with impulsivity, there is an association between these disorders established in the literature (Kessler et al., 2006; Koyuncu et al., 2019). For instance, studies have demonstrated that the co-occurrence of SAD and ADHD is widespread for both adolescents (Chavira et al., 2004; Yüce et al., 2013) and adults (Kessler et al., 2006; Koyuncu et al., 2015; Mörtberg et al., 2012; Safren et al., 2001). Furthermore, a study on children found that five times as many children with ADHD met the diagnostic criteria of more than one anxiety disorder (not necessarily SAD) compared to children without ADHD (Spencer et al., 1999), hence, a more severe total symptom burden. Despite SAD and ADHD being a common comorbidity, the potential links between the two disorders has been underexplored and often overlooked in research (Koyuncu et al., 2018, 2019). One reason for this might be that anxiety disorders (and even internalizing disorders) are often grouped together in scientific studies, that is, results are not always reported separately for each anxiety disorder (Jarrett & Ollendick, 2008; Schatz & Rostain, 2006). This can perhaps be rationalized with the great overlap between different anxiety disorders, for example, generalized anxiety disorder (GAD) and SAD in youth (Whitmore et al., 2013). Nonetheless, these are considered separate diagnoses in the DSM nosology (APA, 2013), meaning that the overlap is not perfect and that it is important to explore nuances between different anxiety disorders when it comes to comorbid ADHD. Another reason for the apparent lack of investigation could be the counterintuitive nature of comorbid ADHD and SAD, which is comprised of individuals who might be impulsive but also shy, and therefore might not live up to either the typically shy or impulsive stereotypes. A third reason might be the fact that much research on ADHD has predominantly centered on children, a group in which social anxiety tends to be less common as it typically emerges during early adolescence (Caouette & Guyer, 2014). This is perhaps partly due to the extensively used parent and teacher ratings with this age group, which may underestimate certain internalizing symptoms in younger children (Schatz & Rostain, 2006). To the best of our knowledge, no previous systematic review has been conducted to comprehensively explore the relationship between SAD and ADHD in adolescents and adults.

Adolescence poses unique challenges for individuals with social anxiety as well as those with ADHD. This is partly because during adolescence, the significance and complexity of peer relationships intensifies (Gardner & Gerdes, 2015), and young people become more aware of their social selves (McKay et al., 2023). Adolescents with ADHD often encounter social challenges due to impulsivity and difficulties in self-regulation, which may lead to behaviors like interrupting others, making inappropriate comments, or struggling to focus. According to *the executive inhibition model* (Barkley, 1997), the lack of cognitive inhibition in ADHD may lead to anxiety through mechanisms such as disruptions in relationships with family and friends (as inhibition is key in social relationships; Schatz & Rostain, 2006). These difficulties may become more pronounced over time “when behavior becomes increasingly reliant on planning and control of cognitive processing” (Schatz & Rostain, 2006, p. 145). According to another model, *the motivational model* (Quay, 1998), “ADHD children do not have normal responses to cues for the consequences of their reward-seeking behavior and that this in turn leads to impulsive, poorly regulated, and socially inappropriate behavior” (Schatz & Rostain, 2006, p. 146). Consequently, adolescents may face an elevated risk of social rejection (Faraone et al., 2021; Koyuncu et al., 2018), and this, in turn, might increase their vulnerability to develop mental health issues, including heightened anxiety (Gardner & Gerdes, 2015; McKay et al., 2023). Adolescence is also a sensitive period for developing social anxiety, as individuals often use social avoidance as a coping mechanism for their anxiety. Nevertheless, as social relationships become increasingly vital during adolescence (Haller et al., 2015), avoiding them can be particularly detrimental, and can help drive a negative spiral leading to increased social anxiety over time.

Given that ADHD is classified as a developmental disorder, the intensity of its symptoms and the way they manifest can vary depending on a person’s age (i.e., age could be seen as a moderator). Furthermore, there are notable individual differences in how these symptoms are displayed and the level of impairment they cause. Although difficulties related to attention typically persist over time, impulsivity tends to be more prevalent in younger individuals and becomes less prominent as they transition into adulthood (Malloy-Diniz et al., 2007). In addition, impulsivity becomes especially prominent during adolescence (Dekkers et al., 2022). This is partly attributed to the ongoing development of brain structures responsible for impulse control, behavioral regulation, and emotional regulation, which lag behind the maturation of the limbic system, responsible for emotional functions. This developmental discrepancy, coupled with experimentation and boundary-testing as teenagers strive for increased independence, may help explain why adolescents display more impulsive and emotion-driven behavior compared to adults (Martel et al., 2016; McKay et al., 2023)—an important reason for why we wanted to explore the relationship between SAD and ADHD in both adolescents and adults.

The primary objective of the current review was to systematically gather empirical data on the link between SAD (diagnostic or subdiagnostic, i.e., not formally assessed or do not meet the diagnostic criteria) and ADHD (diagnostic or subdiagnostic) in both clinical and community populations of adolescents and adults. We aimed to address the following research questions: (1) what is the prevalence of ADHD in adolescents and adults with SAD, (2) what is the prevalence of SAD in adolescents and adults with ADHD, (3) is comorbid SAD and ADHD associated with a higher severity of symptoms, and (4) does the literature on comorbid SAD and ADHD recognize a subtype of SAD high on impulsivity? Answers to these questions will potentially fill some of the previously identified research gaps regarding the relationship between SAD and ADHD, (such as combined prevalence rates of comorbid ADHD and SAD specifically, as opposed to anxiety disorders in general; and to what extent an anxiety-driven impulsive subtype of SAD is documented in studies on comorbid SAD and ADHD), and perhaps identify additional, more nuanced research gaps, paving the way for future studies.

## Method

### Study Design

To address our research questions, a systematic literature review was conducted in line with Hesser and Andersson (2015). The review followed the international PRISMA guidelines for reporting systematic reviews (Page et al., 2021). Compiling a review of all research on the link between social anxiety and ADHD poses several challenges. As previously mentioned, the terminology and diagnostic criteria for both social anxiety and ADHD have undergone significant changes over the years (APA, 1994, 2013). This has led to a wide variation in reported prevalence depending on when different studies were conducted. To reduce the risk of grouping together vastly different disorders, and the fact that most research linking high impulsivity and social anxiety has been conducted since 2000, only studies published from the year 2000 onwards were included in the review. We also only focused on studies with adolescents and adults in both clinical and non-clinical populations, but included the term “teenagers” (as the adolescent age range varies across studies).

### Search Strategy

The first article search was carried out by psychology students WGS and VN during the spring of 2023, and an updated search was conducted by the more experienced SJS (PsyD) on August 4 2023. The article searches were carried out in PsycInfo, PubMed, Scopus and Web of Science. The search terms were “*social anxiety*” *OR* “*social phobia*” *AND* “*attention deficit hyperactivity disorder*” *OR* “*ADHD*,” that is, with Boolean operators. The inclusion criteria were: (1) both “social anxiety” and “ADHD” (or synonymous terms, abbreviations) in the title and/or abstract; (2) both “social anxiety” and “ADHD” (or symptoms of both) in the results; (3) a quantitative research design; and (4) peer reviewed. The exclusion criteria were: (1) language other than English; (2) publication before the year 2000; (3) meta-analysis and/or systematic review, (4) children as participants or children and adolescents combined into a single group; and (5) primarily investigating disorders and phenomena other than “social anxiety” and “ADHD.”

### Selection Process

**Figure 1.**
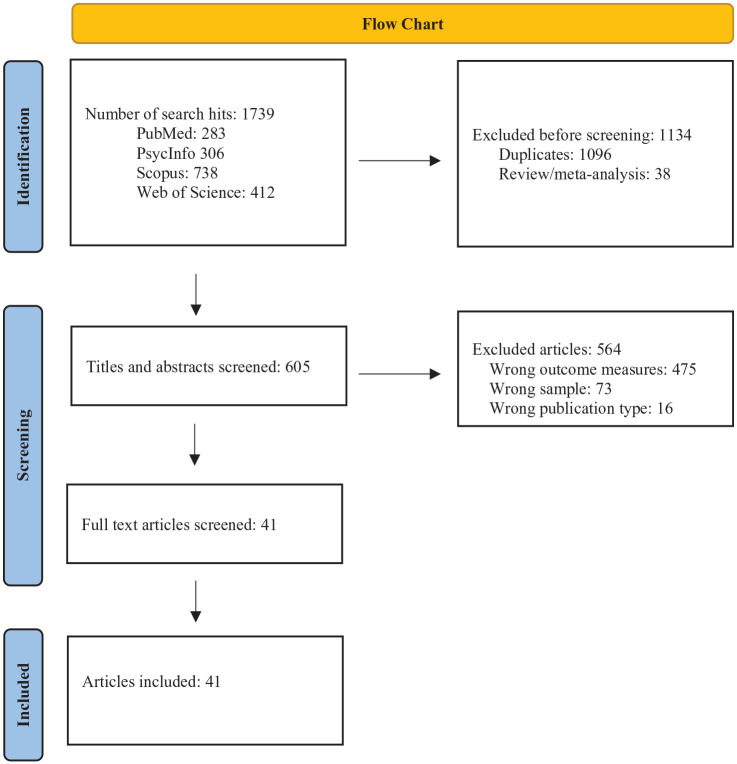
Flow chart of the selection process.

Figure 1 provides an overview of the selection process. As a first step, using filters for publication year and language, the searches resulted in 1,739 hits in total. The web tool Rayyan was used to remove duplicates (1,096) and reviews/meta-analyses (38). As a second step, titles and abstracts were screened, and studies that did not include outcome measures of both social anxiety and ADHD (474), studies including wrong populations (e.g., children), and studies of irrelevant publication type (e.g., study protocols) were excluded. Full-text screening (*n* = 41) were independently conducted by WGS and VN in the first step, before comparison of their ratings. Potential discrepancies were resolved by discussion. In the second step, SJS read the full-text articles and confirmed inclusion of the 41 articles in the final systematic review. Forward searches were conducted via citations in Google Scholar, and backward searches were conducted via the reference lists of all included articles, but no additional relevant articles were found.

### Data Extraction

The following data were extracted from the included articles: total number of participants in the study, number of participants meeting the criteria for ADHD and/or SAD, age range and/or mean age of participants, number of males, outcome measures (rating scales and diagnostic instruments) primarily related to ADHD and/or SAD, and results involving ADHD and social anxiety. These data are presented in tabular form in the Results section. To synthesize the results of quantitative studies, a meta-analytic approach is desirable (Statens beredning för medicinsk och social utvärdering [SBU], 2020). However, this was not possible due to the lack of sufficiently homogeneous methodological and clinical data in the included articles. For example, the studies varied in both participant characteristics and outcome measures. Therefore, the results were synthesized using a narrative approach, which relies on text and words instead of statistical calculations (SBU, 2020).

### Quality Assessment

The quality assessment for the included studies was conducted by SJS and MT using the *Effective Public Health Practice Project Tool* (EPHPP), which assesses the quality of quantitative studies (Thomas et al., 2004). The method involves evaluating nine aspects of empirical studies: (1) selection bias, (2) study design, (3) confounders, (4) blinding, (5) data collection methods, (6) withdrawals and drop-outs, (7) intervention integrity, (8) analyses, and (9) global rating (Thomas et al., 2004). As the majority of the studies included in the current review were cross-sectional in nature and did not include interventions, blinding, intervention integrity, and analyses were excluded from evaluation. Ratings including versus excluding the score respectively for confounders were compared and discussed. Following the recommendation by Thomas et al. (2004), the overall rating was considered weak if two or more aspects were rated as weak, moderate if there was one weak and several moderate aspects, and strong if there were no weak and at least two strong aspects. After individual assessments, three discrepancies in the ratings were identified, and discussions were held to reach consensus on these.

## Results

A total of 41 studies were included in the final review (see Table 1). These studies were conducted in various countries, including the USA (*n* = 14), Turkey (*n* = 8), Brazil (*n* = 2), Korea (*n* = 2), Germany (*n* = 2), Norway (*n* = 1), Taiwan (*n* = 1), the UK (*n* = 1), Australia (*n* = 1), Sweden (*n* = 1), New Zealand (*n* = 1), Japan (*n* = 3), Canada (*n* = 1), the Netherlands (*n* = 1), Iceland (*n* = 1), France (*n* = 1) and Greece (*n* = 1). The majority of the studies had a cross-sectional design (*n* = 33), while the remaining studies were case-control (*n* = 5) or longitudinal studies (*n* = 4). Of the 41 studies, 30 were conducted exclusively with adult participants, 3 studies included both adults and adolescents, and 8 studies focused solely on adolescents. In 28 studies, participants were assessed based on the DSM-IV criteria to determine the presence of SAD and/or ADHD, while three other studies used the DSM-III, the DSM-III-R and the DSM-IV-TR criteria, respectively. The remaining ten studies did not specify whether participants met the diagnostic criteria for any disorder. Additionally, two studies utilized the same sample (Karam et al., 2015, 2017).

**Table 1.** Data Extracted From the Included Studies. The Results Are Reported for Each of the Four Research Questions: (1) What is the Prevalence of ADHD in Adolescents and Adults With SAD?, (2) What is the Prevalence of SAD in Adolescents and Adults With ADHD?, (3) Is Comorbid SAD and ADHD Associated With a Higher Severity of Symptoms?, and (4) Does the Literature on Comorbid SAD and ADHD Recognize a Subtype of SAD High on Impulsivity?

Authors (year); country; purpose; design	Participants	Outcome measures	Results
**Anker et al. (2018)** Norway. Examined the prevalence of comorbid disorders in a clinical sample of adults with ADHD. Cross-sectional study.	ADHD (*n* = 548) according to the DSM-IV. Age range 18–69 years (mean 36.6). Male (*n* = 277).	Social phobia: the MINI International Neuropsychiatric Interview (M.I.N.I).	(1) N/A (2) Diagnostic social phobia was one of the most common comorbid disorders in adults with diagnostic ADHD, with a point prevalence of 14.2% (variable-oriented). (3) Education after high school and work participation were both associated with lower rates of comorbid disorders in general. (4) N/A
**Becker et al. (2015)** The USA. Examined symptoms of anxiety and depression in relation to social skills in a clinical sample of adolescents with ADHD. Cross-sectional study.	ADHD (*n* = 310), of which ADHD-i (*n* = 157) and ADHD-c (*n* = 153) according to the DSM-IV. Age range 10–14 years (mean 12.09). Male (*n* = 219).	ADHD: the Children’s Interview for Psychiatric Syndromes (P-ChIPS) and the Disruptive Behavior Disorder Rating Scale (DBD). Social skills: the Social Skills Improvement System (SSIS) Social acceptance: the Self-Perception Profile for Children (SPPC). Anxiety: the Multidimensional Anxiety Scale for Children (MASC).	(1) N/A (2) N/A (3) Subdiagnostic social anxiety was negatively corelated with self-assessed social skills and both self- and parent rated social acceptance in adolescents with diagnostic ADHD (variable-oriented). ADHD-c was negatively correlated with parent rated social skills, whereas ADHD-I was negatively correlated with self-assessed social acceptance. (4) N/A
**Christian et al. (2021)** The USA. Examined subgroups of undergraduate students based on impulsivity and perfectionism dimensions, and the relation between these and social anxiety (among other psychiatric disorders). Cross-sectional study.	Total (*n* = 1,353) of which ADHD (*n* = 47), anxiety disorder (*n* = 229), depression (*n* = 218), Obsessive-compulsive disorder (*n* = 30), Posttraumatic stress disorder (*n* = 28), eating disorder (*n* = 85), and substance use disorder (*n* = 1) according to the DSM-IV. Age range 16–70 years (mean = 19.93). Male (*n* = 248).	ADHD: the Barkley Adult adhd Rating Scale-IV (BAARS-IV). Social anxiety: the Social Interaction Anxiety Scale (SIAS). Impulsivity: the UPPS-P Impulsive Behavior Scale (UPPS-P). Perfectionism: the Frost Multidimensional Perfectionism Scale (FMPS).	(1) N/A (2) N/A (3) N/A (4) Four subgroups were identified (person-oriented): Perfectionistic/Impulsive, Impulsive, Low both, and Perfectionistic. The Perfectionistic/Impulsive group included half the sample, and was associated with more symptoms of ADHD (subdiagnostic), social anxiety (subdiagnostic), and depression compared to the other groups.
**Coskun et al. (2020)** Turkey. Examined comorbid disorders in a clinical sample of children and adolescents with ADHD. Cross-sectional study.	Total (*n* = 154), of which adolescents (*n* = 75). Total age range 6–17 years. Male adolescents (*n* = 60).	Social anxiety: the K-SADS children-present and lifetime version—Turkish version; the screen for child anxiety related emotional disorders (SCARED)-child form.	(1) N/A (2) The prevalence of lifetime diagnostic social phobia was 8% in adolescents with diagnostic ADHD-i, and 6.67% in adolescents with diagnostic ADHD-c (variable-oriented). (3) N/A (4) N/A
**Edel et al. (2010)** Germany. Examined the interactions between alexithymia, emotion processing, and social anxiety in a clinical sample of adults with ADHD. Cross-sectional study.	ADHD (*n* = 73) of which ADHD-i (*n* = 33) and ADHD-c (*n* = 40) according to the DSM-IV. Age range 18–66 years (mean = 40). Male (*n* = 39).	ADHD: the Wender Utah Rating Scale, German Short version (WURS-K). Social anxiety: the International Diagnostic Checklist for DSM-IV (IDCL); the Social Phobia Scale (SPS); the SIAS. Alexithymia: the Toronto Alexithymia Scale (TAS-20	(1) N/A (2) The point prevalence of diagnostic social phobia was 39.7% in adults with diagnostic ADHD (variable-oriented). (3) About 22% were highly alexithymic, which is closely linked to emotion processing problems (although the number is equivalent to community samples). (4) N/A
**Einarsson et al. (2009)** Iceland. Examined the relation between ADHD symptoms and comorbid disorders in incarcerated male adults. Cross-sectional study.	Total (*n* = 90) of which symptomatic ADHD (*n* = 27), and non-ADHD symptomatic (*n* = 63) according to the DSM-IV. Age range 19–56 years (mean 31). Male (*n* = 90).	ADHD: the WURS; the Diagnostic Statistical Manual-IV Checklist of ADHD symptoms (DSM-IV ADHD Checklist). Social Anxiety: the M.I.N.I.	(1) N/A (2) The prevalence of current diagnostic social phobia was 30% in the subdiagnostic ADHD group, compared to 10% in the non-ADHD symptomatic group (variable-oriented). (3) N/A (4) N/A
**Evren et al. (2017)** Turkey. Examined the relation between symptoms of SAD and probable ADHD in university students, while controlling for symptoms of anxiety, depression and personality traits. Cross-sectional study.	Probable ADHD (*n* = 96) according to the DSM-.IV. Mean age 21.66. Male (*n* = 37). Not ADHD (*n* = 359). Mean age 21.80. Male (*n* = 147).	ADHD: the Adult adhd Self-Report Scale v1.1 (ASRS-v1.1). Social anxiety: the Liebowitz Social Anxiety Scale (LSAS).	(1) N/A (2) N/A (3) The level of subdiagnostic social anxiety symptoms was higher in students with probable ADHD (subdiagnostic) compared to students without ADHD (variable-oriented). The neuroticism personality score was higher in the ADHD group, whereas the extraversion score was the same for both groups. (4) N/A
**Fredrick et al. (2020)** The USA. Examined whether two different forms of attentional difficulties (ADHD and sluggish cognitive tempo—SCT) moderated the relation between fear of social evaluation and social anxiety in university students. Cross-sectional study.	Total (*n* = 4,756). Age range 18–29 years (mean = 19.28). Male (*n* = 1,289).	ADHD: the BAARS-IV. Social anxiety: the SIAS. Fear of negative evaluation: The Brief Fear of Negative Evaluation Scale (BFNE). Fear of positive evaluation: The Fear of Positive Evaluation Scale (FPES).	(1) N/A (2) N/A (3) Sluggish cognitive tempo, but not subdiagnostic ADHD intensified the relation between fears of social evaluation and subdiagnostic social anxiety (variable-oriented). (4) N/A
**Gorlin et al. (2016)** The USA. Compared prevalence rates of the most common psychiatric disorders in a clinical sample of adults with and without ADHD. Cross-sectional study.	ADHD (*n* = 204) of which ADHD-i (*n* = 87), ADHD-h (*n* = 18), and ADHD-c (*n* = 99) according to the DSM-IV. Mean age 34.9. Male (*n* = 102). Not ADHD (*n* = 929). Mean age = 41.1. Male (*n* = 374).	ADHD: the Structured Clinical Interview for DSM Disorders (SCID) Social phobia: the SCID	(1) N/A (2) The point prevalence of diagnostic social phobia was higher in patients with diagnostic ADHD (38.2%) compared to patients without ADHD (28.7%). The prevalence of diagnostic social phobia was higher in the ADHD-i subtype (42.5%) compared to ADHD-c (34.3%; variable-oriented). (3) N/A (4) N/A
**Greenberg and De Los Reyes (2022)** The USA. Examined whether adolescents with symptoms of both social anxiety and ADHD have more impaired social functioning compared to adolescents with only social anxiety or only ADHD in a mixed-clinical/community control sample. Case-control study.	Total (*n* = 134) of which clinical disorder (*n* = 45) according to the DSM-IV, and no clinical disorder (*n* = 89). Age range 14–15 years (mean = 14.5). Male (*n* = 45).	ADHD: the Adult adhd Self-Report Scale, six item scale (ASRS-6). Social anxiety: the Social Phobia and Anxiety Inventory for Children (SPAIC). Peer related impairments: Unfamiliar peer paradigm tasks. Social skills: Unfamiliar peer paradigm tasks.	(1) N/A (2) N/A (3) Four groups were identified: Low social anxiety/Low ADHD, High social anxiety/Low ADHD, Low social anxiety/High ADHD, and High social anxiety/High ADHD (person-oriented). The High social anxiety/High ADHD group (subdiagnostic on both) had more peer related difficulties and less social skills compared to the other three groups. (4) N/A
**Kajitani et al. (2019)** Japan. Examined the association between the culture-specific taijin kyofusho and ADHD and SAD and ADHD in university students. Cross-sectional study.	Total (*n* = 673). Mean age 21.1. Male (*n* = 500).	ADHD: the ASRS version 1.1. Social anxiety: the LSAS Japanese version; the Taijin-Kyofu-sho scale (TKs).	(1) There was a moderate positive correlation between subdiagnostic ADHD and taijin kyofusho, compared to a weak correlation between subdiagnostic ADHD and subdiagnostic social anxiety (premature person-oriented). (2) N/A (3) N/A (4) N/A
**Kajitani et al. (2021)** Japan. Examined ADHD traits in university students with SAD or the culture-specific taijin kyofusho . Cross-sectional study.	Total (*n* = 818) of which social anxiety (*n* = 86), taijin kyofusho (*n* = 122), and the control group (*n* = 629). Age range 18–40 years (mean 21.4). Male (*n* = 628).	ADHD: the ASRS version 1.1. Social anxiety: the LSAS; the Taijin-Kyofu-sho scale (TKs).	(1) The prevalence of subdiagnostic ADHD was higher in students with subdiagnostic social anxiety (45.3%) and taijin kyofusho (32.7%) compared to the control group (18.8%; premature person-oriented). (2) N/A (3) N/A (4) N/A
**Karam et al. (2015)** Brazil. Examined predictors of persistent ADHD in a clinical sample of adults with ADHD vs. remission 7 years after baseline. Longitudinal study.	Timepoint 1: ADHD (*n* = 344) of which ADHD-i (*n* = 132), ADHD-h (*n* = 20), and ADHD-c (*n* = 192) according to the DSM-IV. Age range18–68 years (mean = 34.1). Male (*n* = 172). Timepoint 2: ADHD (*n* = 225). Age range 23–68 years (mean = 42). Male (*n* = 108).	ADHD: the Kiddie Schedule for Affective Disorders and Schizophrenia: Epidemiological version (K-SADS-E). Social phobia: Structured Clinical Interview for DSM -IV (SCID-IV).	(1) N/A (2) N/A (3) More symptoms of inattention, more symptoms of hyperactivity/impulsivity, and more symptoms of diagnostic social phobia all predicted persistent diagnostic ADHD (varible-oriented). (4) N/A
**Karam et al. (2017)** Brazil. Examined directions and predictors of changes for each ADHD domain in a clinical sample of adults with ADHD. Longitudinal study.	Timepoint 1: ADHD (*n* = 344) of which ADHD-i (*n* = 132), ADHD-h (*n* = 20), and ADHD-c (*n* = 192) according to the DSM-IV. Age range 18–68 years (mean = 34.1). Male (*n* = 172). Timepoint 2: ADHD (*n* = 225). Age range 23–68 years (mean = 42). Male (*n* = 108).	ADHD: the K-SADS-E. Social phobia: the SCID-IV.	(1) N/A (2) N/A (3) Diagnostic social phobia was associated with less reduction in symptoms of inattention between the first and second timepoints which contributed to the persistence of diagnostic ADHD symptoms (variable-oriented). (4) N/A
**Koyuncu et al. (2017)** Turkey. Examined the relation between childhood ADHD and interpersonal sensitivity in a clinical sample of adults with social anxiety. Cross-sectional study.	Social anxiety (*n* = 125) according to the DSM-IV. Age range 18–65 years (mean = 29.1). Male (*n* = 62).	ADHD: the Kiddie Schedule for Affective Disorders and Schizophrenia, Present and Lifetime version (K-SADS-PL). Social anxiety: the Structured Clinical Interview for DSM Disorders, clinical version (SCID-I/CV); the LSAS Sensitivity for non-verbal cues: the Interpersonal Sensitivity Measure (IPSM).	(1) N/A (2) N/A (3) Comorbid ADHD and SAD (both diagnostic) was associated with higher interpersonal sensitivity compared to social anxiety without ADHD (variable-oriented). (4) N/A
**Koyuncu et al. (2019)** Turkey. Examined the impacts of the inattentive and the combined subtypes of childhood ADHD in a clinical sample of adults with social anxiety. Cross-sectional study.	Social anxiety (*n* = 142) according to the DSM-IV. Age range 18–65 years (mean = 28.13). Male (*n* = 78).	ADHD: the K-SADS-PL Social anxiety: the SCID-I/CV; the LSAS.	(1) The prevalence of childhood ADHD (diagnostic) was 62% in adults with SAD (diagnostic). The ADHD-I subtype was associated with symptoms of social anxiety as well as an earlier debut of social anxiety compared to the ADHD-c subtype (variable-oriented). (2) N/A (3) N/A (4) N/A
**Koyuncu et al. (2015)** Turkey. Examined the prevalence of childhood ADHD in a clinical sample of adults with social anxiety. Cross-sectional study.	Social anxiety (*n* = 130) according to the DSM-IV. Age range 18–65 years (mean = 29.59). Male (*n* = 78).	ADHD: the K-SADS-PL. Social anxiety: the SCID-I/CV; the LSAS. Level of functioning: the Global Assessment of Functioning (GAF).	(1) The prevalence of childhood ADHD (diagnostic) was 72.3% in adults with SAD (diagnostic). ADHD-i was the most common subtype (variable-oriented). (2) N/A (3) Comorbid ADHD and social anxiety was associated with more symptoms of social anxiety and a lower degree of functioning. The lifetime prevalence of another psychiatric disorder was higher in the comorbid ADHD and social anxiety group compared to social anxiety without ADHD. (4) N/A
**Lipton et al. (2016)** The USA. Examined differences among profiles of social anxiety and impulsivity regarding externalizing behaviors in college students. Cross-sectional study.	Total (*n* = 375). Mean age 19.63. Male (*n* = 86).	ADHD: the ASRS; UPPS-P. Social anxiety: the SPS; the SIAS; the Liebowitz Social Anxiety Scale-Self Report (LSAS-SR).	(1) N/A (2) N/A (3) N/A (4) Three profiles were identified (person-oriented): Low social anxiety/Low impulsivity (74%), High social anxiety/Low impulsivity (17%), and High social anxiety/High impulsivity (8.5%). The High social anxiety/High impulsivity (subdiagnotic) group reported more ADHD symptoms and more externalizing behaviors compared to the other groups.
**Liu et al. (2014)** Taiwan. Examined the relation between symptoms of ADHD and symptoms of anxiety (including social anxiety) in a community-based sample of adolescents. Cross-sectional study.	Total (*n* = 4 716) of which ADHD symptoms (*n* = 653) and no ADHD symptoms (*n* = 4 063) according to the DSM-IV. Age range 12–17 years. Male (*n* = 2 184).	ADHD: the ASRS Social anxiety: the Multidimensional Anxiety Scale for Children Taiwanese version (MASC-T).	(1) N/A (2) N/A (3) Subdiagnostic ADHD was associated with more severe subdiagnostic social anxiety compared to those without ADHD (variable-oriented). (4) N/A
**Marmorstein (2006)** The USA. Examined patterns of comorbid disorders associated with social phobia in a community-based sample of adolescents. Cross-sectional study.	Total (*n* = 1,295) of which performance specific social phobia (*n* = 34) and generalized social phobia (*n* = 56) according to the DSM-III-R. Age range 9–17 years. Male (*n* = 686).	ADHD: the DISC Personality Test (DISC). Social phobia: the DISC.	(1) The generalized social phobia subtype (diagnostic) was associated with diagnostic ADHD, whereas the performance-focused subtype was not (variable-oriented). (2) N/A (3) N/A (4) N/A
**Marmorstein (2007)** The USA. Examined the relation between anxiety and externalizing disorders in a community-based sample of adolescents. Cross-sectional study.	Total (*n* = 1,304). Age range 9–18 years. Male (*n* = 682).	ADHD: the DISC. Social phobia: the DISC.	(1) N/A (2) Diagnostic ADHD was positively correlated with diagnostic social phobia (variable-oriented). (3) N/A (4) N/A
**Michelini et al. (2015)** The UK. Examined the relation between subtypes of anxiety and ADHD symptoms in a community-based sample of adolescent siblings and twins. Cross-sectional study.	Total (*n* = 1,564). Age range 14–23 years (mean = 17). Male (*n* = 649).	ADHD: the Youth Self-Report (YSR); Young Adult Self Report (YASR). Social anxiety: the Spence Children’s Anxiety Scale (SCAS),	(1) N/A (2) N/A (3) N/A (4) Subdiagnostic social anxiety was positively correlated with inattentive symptoms of ADHD (subdiagnostic), but not with hyperactivity/impulsivity (variable-oriented).
**Moore et al. (2016)** Australia. Examined the prevalence of comorbid disorders in a clinical sample of incarcerated adults with ADHD. Cross-sectional study.	Total (*n* = 88) of which ADHD (*n* = 15) according to the DSM-IV, and not ADHD (*n* = 73). Age range 18–72 years (mean 41). Male (*n* = 67).	ADHD: the ASRS; the M.I.N.I. Plus. Social phobia: the M.I.N.I. Plus.	(1) N/A (2) The point prevalence of diagnostic social phobia was higher in prisoners with diagnostic ADHD (46.7%) compared to those without ADHD (15.1%; variable-oriented). (3) N/A (4) N/A
**Mörtberg et al. (2012)** Sweden. Examined the prevalence of ADHD symptoms in a clinical sample of adults with social phobia. Longitudinal study.	ADHD (*n* = 178) according to the DSM-IV. Age range 16–57 years (mean = 31). Male (*n* = 89). Social phobia (*n* = 39) according to the DSM-IV. Age range 21–62 years (mean = 35.7). Male (*n* = 14). Comorbid disorders (*n* = 88). Age range 18–56 years (mean = 32.3). Male (*n* = 47).	ADHD: the Wender Utah Rating Scale (WURS); the ASRS. Social anxiety: the LSAS-SR.	(1) The prevalence of childhood ADHD (diagnostic) was 7.8% and the prevalence of adult ADHD was 5.1% in adults with diagnostic social phobia, similar to the prevalence of ADHD in the general population (variable-oriented). (2) N/A (3) N/A (4) N/A
**O’Rourke et al. (2020)** The USA. Examined anxiety symptoms and disorders in a clinical sample of students with ADHD compared to students without ADHD. Case-control study.	ADHD (*n* = 46) of which ADHD-i (*n* = 23), ADHD-c (*n* = 20) and unspecified ADHD (*n* = 3) according to the DSM-IV-TR. Mean age 20.5. Male (*n* = 15). Not ADHD (*n* = 46). Mean age 20. Male (*n* = 16).	ADHD: the Adhd Rating Scale IV (adhd-RS-IV); the Conners’ Adult adhd Rating Scales—Self Report: Long Version (CAARS-S:L). Social anxiety: the LSAS-SR. Anxiety disorders: the SCID-CV.	(1) N/A (2) The prevalence of diagnostic social phobia was 9% in students with diagnostic ADHD compared to 11% in the comparison group (variable-oriented). (3) N/A (4) N/A.
**Park et al. (2011)** Korea. Examined the prevalence, correlates, and comorbid disorders in adults with ADHD symptoms. Cross-sectional study.	ADHD symptoms (*n* = 69) according to the DSM-IV. Age range 18–59 years. Male (*n* = 41). No ADHD symptoms (*n* = 6,012). Age range 18–59 years Male (*n* = 3,036).	ADHD: the ASRS. Social phobia: the Composite International Diagnostic Interview Korean version (K-CIDI).	(1) N/A (2) The lifetime prevalence of diagnostic social phobia was higher 2.9% in adults with subdiagnostic ADHD (2.9%), compared to adults without ADHD symptoms (0.5%; variable-oriented). (3) N/A (4) N/A
**Pehlivanidis et al. (2020)** Greece. Examined comorbid disorders in adults with ADHD. Cross-sectional study.	ADHD (*n* = 151). Mean age 31. Male (*n* = 106).	ADHD: the Barkley Adult ADHD Rating Scale-IV (BAARS-IV). Social anxiety: the M.I.N.I.	(1) N/A (2) The prevalence of diagnostic social phobia was 3.3% in adults with diagnostic ADHD (variable-oriented). (3) N/A (4) N/A
**Peyre et al. (2022)** France. To examine whether an earlier onset of SAD was associated with more comorbid disorders in adults in a community sample. Cross-sectional study.	Total (*n* = 34,604). Onset of SAD before 12 years (*n* = 658), according to the DSM-IV Mean age 44.4. Male 40%. Onset of SAD between 12 and 17 years (*n* = 663). Mean age 44.4. Male 43.8%. Onset of SAD between 18 and 39 years (*n* = 663). Mean age 39.0. Male 38.7% Onset of SAD between 40 and 86.5 years (*n* = 415). Mean age 56.1. Male 39%. No history of SAD (*n* = 32,205). Mean age 48.4. Male 49.5%.	ADHD: the Alcohol Use Disorder and Associated Disabilities Interview Schedule, DSM-IV version (AUDADIS-IV). Social anxiety: AUDADIS-IV.	(1) The prevalence of diagnostic ADHD was 1.4% in adults with onset of diagnostic SAD before 12 years, 1.1% in adults with onset of SAD between 12 and 17 years, 1.3% in adults with onset of SAD between 18 and 39 years, 1.6% in adults with onset of SAD between 40 and 86.5 years, and 0.1% in adults with no history of SAD (variable-oriented). (2) N/A (3) N/A (4) N/A
**Pine et al. (2000)** The USA. Examined the influence of social phobia and ADHD symptoms on symptoms of conduct disorder over time in a community-based sample of adolescents. Longitudinal study.	Timepoint 1: Total (*n* = 776) of which ADHD (*n* = 93), social phobia (*n* = 65), severe depression (*n* = 25), specific phobia (*n* = 90), generalized anxiety disorder (*n* = 111), separation anxiety (*n* = 67) and conduct disorder (*n* = 89) according to the DSM III. Age range 9–18 years (mean = 13.7). Male (*n* = 388). Timepoint 2: Total (*n* = 760) of which ADHD (*n* = 59), social phobia (*n* = 74), severe depression (*n* = 22), specific phobia (*n* = 45), generalized anxiety disorder (*n* = 61), separation anxiety (*n* = 28), and conduct disorder (*n* = 70) according to DSM-III-R. Age range 11–20 years (mean = 16.4). Male (*n* = 380). Timepoint 3: Total (*n* = 716) of which ADHD (*n* = 8), social phobia (*n* = 40), severe depression (*n* = 59), specific phobia (*n* = 159), generalized anxiety disorder (*n* = 36), and conduct disorder (*n* = 48) according to the DSM-III-R. Age range 17–26 years (mean = 22.1). Male (*n* = 358).	ADHD: the DISC. Social phobia: the DISC.	(1) N/A (2) N/A (3) Low levels of social phobia symptoms (diagnostic) and high levels of ADHD symptoms (diagnostic) had the strongest correlations with conduct disorder scores over time (variable-oriented). (4) N/A
**Rucklidge et al. (2016)** New Zealand. Examined rates of comorbid disorders in a clinical sample of adults with ADHD compared to a community control group. Case-control study.	ADHD (*n* = 158) according to the DSM-IV. Mean age 33.48. Male (*n* = 103). Not ADHD (*n* = 64). Mean age 30.47. Male (*n* = 35).	ADHD: the Conners’ Adult adhd Rating Scales (CAARS); the Conners Adult adhd Diagnostic Interview for DSM-IV (CAADID). Social phobia: the SCID.I.	(1) N/A (2) Diagnostic social phobia was more common in adults with diagnostic ADHD (31%) compared to the control group (11%; variable-oriented). (3) N/A (4) N/A
**Safren et al. (2001)** The USA. Examined the prevalence of childhood ADHD in a clinical sample of adults with social phobia or generalized anxiety disorder. Cross-sectional study.	Social phobia (*n* = 33) according to the DSM-IV. Age18+. Male (*n* = 13). Generalized anxiety disorder (*n* = 22) according to DSM-IV. Age 18+. Male (*n* = 11).	ADHD: the SCID-IV; the Schedule for Affective Disorders, and Schizophrenia-Lifetime (SADS-L). Social phobia: the SCID-IV; the SADS-L; the Clinical Global Impressions Scale (CGI-S).	(1) The prevalence of childhood ADHD (continuum subdiagnostic—diagnostic) was low in persons with diagnostic social phobia (3%) compared to persons with generalized anxiety disorder (32%). The one patient with comorbid social phobia and childhood ADHD had current generalized anxiety disorder as well (variable-oriented). (2) N/A (3) N/A (4) N/A
**Secnik et al. (2005)** The USA. Examined the prevalence of comorbid disorders in a clinical sample of adults with ADHD. Cross-sectional study.	Total (*n* = 4,505) of which ADHD (*n* = 2,252) and control group (*n* = 2,252). Mean age 31.85. Male 64.30%.	ADHD and social anxiety: Inpatient or outpatient diagnoses in databases according to the ICD-9.	(1) N/A (2) The prevalence of diagnostic social phobia was 0.04% in adults with diagnostic ADHD, compared to 0% in the control group (variable-oriented). (3) N/A (4) N/A
**Thoma et al. (2020)** Germany. Examined social problem solving strategies in a clinical sample of adults with ADHD compared with healthy controls. Case-control study.	ADHD (*n* = 19) according to the DSM-IV. Mean age 36.2. Male (*n* = 9). Not ADHD (*n* = 20). Mean age 36.7. Male (*n* = 10).	Social anxiety: the SPS; the SIAS . Social cognition: the Mentalistic Interpretation Task; the Social Problems Resolution Task; the Social Problem Fluency Task.	(1) N/A (2) N/A (3) Adults with diagnostic ADHD did not differ from healthy controls regarding the Mentalistic Interpretation Task or the Social Problems Resolution Task, but they did have fewer optimal solutions on the Social Problem Solving Fluency Task compared to people without ADHD. Persons with ADHD had higher subdiagnostic social interaction anxiety relative to controls (variable-oriented). (4) N/A
**Umeda et al. (2021)** Japan. Examined comorbid disorders in a clinical sample of adults with autism or ADHD. Cross-sectional study.	Total (*n* = 2,450) of which respondents assessed for ADHD (*n* = 2,297) according to the DSM-IV. Age range 20–75 years.	ADHD: the ASRS. Social phobia and hikikomori: the World Mental Health Composite International Diagnostic Interview (WMH-CIDI).	(1) N/A (2) In adults with subdiagnostic ADHD, the lifetime prevalence of subdiagnostic social phobia was 8% and the lifetime prevalence of hikikomori was 9.7% (variable-oriented). (3) N/A (4) N/A
**Umutlu et al. (2022)** Turkey. Examined the level of empathy in a clinical sample of adults with social anxiety, with or without comorbid ADHD. Cross-sectional study.	Comorbid ADHD and social anxiety (*n* = 32) of which ADHD-i (*n* = 21) and ADHD-c (*n* = 11) according to the DSM-IV. Mean age 27.1). Male (*n* = 20). Social anxiety without ADHD (*n* = 40). Mean age 26.4). Male (*n* = 17).	ADHD: the K-SADS-PL; the WURS; the Turgay’s Adult Attention-Deficit /Hyperactivity Disorder (ADD/ADHD) DSM-IV-Based Diagnostic Screening and Rating Scale. Social anxiety: the LSAS. Empathy: the Empathy Quotient (EQ).	(1) The prevalence of diagnostic ADHD was 40.1% in persons with diagnostic social phobia. ADHD-I was the most common subtype (variable-oriented). (2) N/A) (3) Comorbid ADHD and social anxiety was correlated with lower levels of empathy compared to social anxiety without ADHD (p = .014). Lower levels of empathy were correlated with symptoms of both inattention and hyperactivity/impulsivity. (4) N/A
**Van Ameringen et al. (2011)** Canada Examined the prevalence of ADHD in a clinical sample of adults with anxiety disorders. Cross-sectional study.	Anxiety disorder and ADHD (*n* = 36) according to the DSM-IV. Mean age 33.2. Male (*n* = 17). Anxiety disorder without ADHD (*n* = 93). Mean age 33. Male (*n* = 30).	ADHD: the MINI International Neuropsychiatric Interview, Extended version (M.I.N.I Plus); the ASRS. Social anxiety: the SCID; the LSAS.	(1) Diagnostic social phobia was one of the most common comorbid disorders associated with diagnostic ADHD (38.5%; variable-oriented). (2) N/A (3) N/A (4) N/A
**Vogel et al. (2018)** The Netherlands. Examined whether an increased number of ADHD symptoms was associated with higher comorbidity in adults. Cross-sectional data.	Total (*n* = 5,303), of which four or more ADHD symptoms (*n* = 267), three ADHD symptoms (*n* = 427), one-two ADHD symptoms (*n* = 2,201), and no ADHD symptoms (*n* = 2,408). Age range 18–64 years. Male 50.5%.	ADHD: the ASRS version 1.1. Social anxiety: the Composite International Diagnostic Interview.	(1) N/A (2) The prevalence of subdiagnostic social phobia was 8.98% in adults with four or more symptoms of subdiagnostic ADHD, compared to 5.26% for three ADHD symptoms, 1.99 for one or two ADHD symptoms, and 1.55% for no ADHD symptoms (variable-oriented). (3) N/A (4) N/A
**Wilens et al. (2005)** The USA Examined comorbid disorders in a clinical sample of adults with ADHD, and/or substance use disorder. Case-control study.	ADHD (*n* = 20) according to the DSM-IV. Mean age 44.5. Male (*n* = 3). Substance use disorder (*n* = 20) according to the DSM-IV. Mean age 42.6). Male (*n* = 5). ADHD and substance use disorder (*n* = 19). Mean age 44.1. Male (*n* = 8). No diagnosis (*n* = 19). Mean age 42.2. Male (*n* = 2).	ADHD: the SCID; the K-SADS-E. Social phobia: the SCID; the K-SADS-E.	(1) N/A (2) The lifetime prevalence of diagnostic social phobia was 20% in adults with diagnostic ADHD, and 42% in adults with both ADHD and substance use disorder (variable-oriented). (3) N/A (4) N/A
**Yeom et al. (2020)** Korea Examined comorbid disorders in a clinical sample of male military servicemen with ADHD. Cross-sectional study.	ADHD (*n* = 96) according to the DSM-IV. Mean age 21.4. Male (*n* = 96). Not ADHD (*n* = 3,345). Mean age 21.2. Male (*n* = 3,345).	ADHD: the ASRS. Social anxiety: the SIAS.	(1) N/A (2) The point prevalence of subdiagnostic social anxiety was higher in military servicemen with subdiagnostic ADHD (49.5%) compared to those without ADHD (2.6%; variable-oriented). (3) N/A (4) N/A
**Yoldas et al. (2019)** Turkey Compared generalized social anxiety with and without ADHD in terms of avoidant personality disorder in a clinical sample of adults. Cross-sectional study.	Generalized social anxiety (*n* = 42) according to the DSM-IV. Mean age 25.5. Male (*n* = 13). Generalized social anxiety and ADHD(*n* = 34). Mean age 25.3. Male (*n* = 17).	ADHD: the SCID-I; the K-SADS-PL; the Turgay’s Adult Attention-Deficit/Hyperactivity Disorder (ADD/ADHD) DSM-IV-Based Diagnostic Screening and Rating Scale; the WURS. Social anxiety: the SCID-I; the K-SADS-PL; the LSAS.	(1) In adults with generalized social anxiety (diagnostic), the prevalence of childhood ADHD (diagnostic) was 58.2% and the prevalence of adult ADHD was 43% (variable-oriented). (2) N/A (3) Avoidant personality disorder was more common in the comorbid generalized social anxiety and ADHD group compared to those without ADHD. Inattention symptoms of adult ADHD was not associated with the severity of avoidant personality disorder. (4) N/A
**Yüce et al. (2013)** Turkey Examined the distribution of comorbid disorders in a clinical sample of adolescents with ADHD. Cross-sectional study.	ADHD (*n* = 108) of which ADHD-i (*n* = 15) and ADHD-c (*n* = 93) according to the DSM-IV. Age range 6–18 years (mean = 10.26). Male (*n* = 83).	ADHD: the K-SADS-PL. Social phobia: the K-SADS-PL.	(1) N/A (2) The prevalence of diagnostic social phobia was 34.2% in adolescents with diagnostic ADHD (compared to 10% in children). The inattentive subtype was more common (60%) compared to the combined subtype (11.8%; variable-oriented). (3) N/A (4) N/A

*Note.* ADHD = Attention-Deficit/Hyperactivity Disorder; ADHD-c = ADHD combined subtype; ADHD-i = ADHD inattentive subtype; ADHD-h = ADHD hyperactive and impulsive subtype; N/A = not applicable.

### Quality Assessment

In line with previous studies (e.g., Spain et al., 2018), the quality assessment of the included studies was conducted using the EPHPP assessment tool with ratings of weak, moderate, and strong (see Table 2). In several studies, participants were recruited from psychiatric clinics or outpatient settings where they had sought help on their own, increasing the risk of potential selection bias and resulting in lower ratings in this regard. Regarding study design, the majority of the included studies were cross-sectional, with a few exceptions being case-control and longitudinal studies, which also resulted in in weak ratings for the most part. Two or more groups were used across several studies, such as one group of persons with ADHD and another non-clinical control group. In some cases, significant differences between groups were observed in terms of various confounders, such as sex, age, and ethnicity. In two of these studies, confounders were controlled for through stratified sampling, matching, or some form of statistical analysis, leading to higher ratings (“strong”), but in the rest of the studies on this topic it was unclear whether relevant confounders were controlled for, resulting in lower ratings (“weak”). Since strong = no weak ratings, moderate = one weak rating, weak = two or more weak ratings, and the scores on the confounder variable affected the overall ratings to such a great extent, we also wanted to see what the overall ratings would be without this variable.

**Table 2.** Quality Assessment of the Included Studies.

Authors (year)	Selection bias	Study design	Confounders	Method för data collection	Attrition	Overall
Anker et al. (2018)	M	W	W	S	N/A	W
Becker et al. (2015)	W	W	W	S	N/A	W
Christian et al. (2021)	W	W	W	S	N/A	W
Coskun et al. (2020)	W	W	W	M	N/A	W
Edel et al. (2010)	W	W	W	S	N/A	W
Einarsson et al. (2009)	W	W	W	S	N/A	W
Evren et al. (2017)	S	W	W	S	N/A	W
Fredrick et al. (2020)	W	W	W	S	N/A	W
Gorlin et al. (2016)	W	W	W	S	N/A	W
Greenberg and De Los Reyes (2022)	W	M	W	S	N/A	W
Kajitani et al. (2019)	W	W	W	M	N/A	W
Kajitani et al. (2021)	W	W	W	M	N/A	W
Karam et al. (2015)	M	M	W	S	M	M
Karam et al. (2017)	M	M	W	S	N/A	M
Koyuncu et al. (2017)	W	W	W	S	N/A	W
Koyuncu et al. (2019)	W	W	W	S	N/A	W
Koyuncu et al. (2015)	W	W	W	M	N/A	W
Lipton et al. (2016)	W	W	W	S	N/A	W
Liu et al. (2014)	S	W	W	S	N/A	W
Marmorstein (2006)	S	W	W	S	N/A	W
Marmorstein (2007)	S	W	W	S	N/A	W
Michelini et al. (2015)	M	W	W	M	N/A	W
Moore et al. (2016)	M	W	W	M	N/A	W
Mörtberg et al. (2012)	W	M	W	S	N/A	W
O’Rourke et al. (2020)	W	M	W	S	N/A	W
Park et al. (2011)	S	W	W	S	N/A	W
Pehlivanidis et al. (2020)	W	M	W	M	N/A	W
Peyre et al. (2022)	W	M	W	S	N/A	W
Pine et al. (2000)	S	W	S	S	N/A	W
Rucklidge et al. (2016)	W	M	W	S	N/A	W
Safren et al. (2001)	W	M	W	M	N/A	W
Secnik et al. (2005)	W	M	W	W	N/A	W
Thoma et al. (2020)	W	M	W	M	N/A	W
Umeda et al. (2021)	W	W	S	S	N/A	W
Umutlu et al. (2022)	W	M	W	M	N/A	W
Van Ameringen et al. (2011)	W	W	W	M	N/A	W
Vogel et al. (2018)	M	W	W	S	N/A	W
Wilens et al. (2005)	W	M	W	S	N/A	W
Yeom et al. (2020)	S	W	W	M	N/A	W
Yoldas et al. (2019)	W	M	W	M	N/A	W
Yüce et al. (2013)	W	W	W	S	N/A	W

*Note*. S = strong; M = moderate; W = weak; N/A = not applicable.

Data collection methods and measurement instruments varied among the studies. Assessment of dropouts was not applicable for the majority of the studies as both cross-sectional and case-control studies only involved a single measurement. Most studies were judged to be of an overall weak quality, except for two studies which were rated to be of moderate quality, but as the EPHPP assessment tool is designed to evaluate intervention studies (meaning that the design of the included studies produced likely consistently lower ratings), none of these studies were excluded from the review. The ratings excluding the confounder variable resulted in 2 studies of strong quality, 22 studies of moderate quality, and 17 studies of weak quality (see Table S1 in the Supplemental Material), that is, higher quality ratings for 24 out of 41 studies.

### What Is the Prevalence of ADHD in Adolescents and Adults With SAD?

Eleven of the included studies investigated the prevalence of ADHD in adolescents and adults with SAD. Kajitani et al. (2019) found a moderate positive correlation between the culture-specific taijin kyofusho and subdiagnostic ADHD, and a weak positive correlation between subdiagnostic social anxiety and ADHD, in university students. In another study, the prevalence of subdiagnostic ADHD was 45.3% in university students with subdiagnostic social anxiety, compared to 32.7% for taijin kyofusho, and 18.8% for no subdiagnostic social anxiety (Kajitani et al., 2021). Studies that investigated diagnostic ADHD in adults with diagnostic SAD reported prevalence rates of 1.1% (Peyre et al., 2022), 3% (Safren et al., 2001), 7.8% (Mörtberg et al., 2012), 38.5% (Van Ameringen et al., 2011), 40.1% (Umutlu et al., 2022), 58.2% (Yoldas et al., 2019), 62% (Koyuncu et al., 2019), and 72.3% (Koyuncu et al., 2015). All the forementioned studies comprised clinical samples. Only one study included adolescents, in a community-based sample (Marmorstein, 2006). This study found that generalized diagnostic social phobia was associated with diagnostic ADHD (performance-focused social phobia was, however, not associated with ADHD). All the 11 studies were rated weak in terms of overall quality ratings. In three of the studies, the inattentive form of ADHD was most common in adults with diagnostic SAD (Koyuncu et al., 2015, 2019; Umutlu et al., 2022), while the other studies did not differentiate between different forms of ADHD. In summary, the prevalence of diagnostic ADHD in adults and adolescents with diagnostic social anxiety ranged from 1.1 to 72.3% across the reviewed studies.

### What Is the Prevalence of SAD in Adolescents and Adults With ADHD?

Seventeen studies investigated the prevalence of social anxiety in adolescents and adults with ADHD. Of these, only three studies were conducted with adolescents (Coskun et al., 2020; Marmorstein, 2007; Yüce et al., 2013), the rest with adults. Studies that investigated diagnostic SAD in adolescents and adults with diagnostic ADHD (in clinical samples) reported prevalence rates of .04% (Secnik et al., 2005), 3.3% (Pehlivanidis et al., 2020), 9% (O’Rourke et al., 2020), 14.2% (Anker et al., 2018), 14.66% (Coskun et al., 2020), 20% (Wilens et al., 2005), 31% (Rucklidge et al., 2016), 34.2% (Yüce et al., 2013), 38.2% (Gorlin et al., 2016), 39.7% (Edel et al., 2010), and 46.7% (Moore et al., 2016). Studies that investigated diagnostic SAD in adults with subdiagnostic ADHD reported prevalence rates of 2.9% (community sample: Park et al., 2011) and 30% (incarcerated adults: Einarsson et al., 2009). Studies that investigated subdiagnostic SAD in adults with subdiagnostic ADHD reported prevalence rates of 8.98 in a community sample (Vogel et al., 2018) and rates between 8% (Umeda et al., 2021) and 49.5% (Yeom et al., 2020) in clinical samples. Umeda et al. (2021) also examined the culture-specific hikikomori (social withdrawal) and reported a prevalence of 9.7% in adults with diagnostic ADHD (compared to 1.5% in adults without ADHD). Three studies reported that social anxiety was more common for individuals with the inattentive form of ADHD than the combined form (Coskun et al., 2020; Gorlin et al., 2016; Yüce et al., 2013)—all three studies included clinical samples of adults. In sum, prevalence ratings of SAD/subdiagnostic social anxiety in adolescents and adults with diagnostic/subdiagnostic ADHD ranged from 0.4% to 46.7% across the reviewed studies.

### Is Comorbid SAD and ADHD Associated With a Higher Severity of Symptoms?

Fifteen studies addressed whether the comorbidity of social anxiety and ADHD is associated with the severity of SAD or ADHD. Four of these were conducted with adolescents (Becker et al., 2015; Greenberg & De Los Reyes, 2022; Liu et al., 2014; Pine et al., 2000)—two with clinical samples, and two with community-based samples. One study was conducted with both adolescents and adults (Michelini et al., 2015), and the rest with adults only. ADHD was associated with different aspects of social functioning across the studies. For instance, Thoma et al. (2020) showed that adults with diagnostic ADHD (clinical sample) generated significantly fewer optimal solutions on tests measuring social problem-solving. Fredrick et al. (2020) found that inattention in ADHD in university students was correlated with higher self-reported fear of both negative and positive evaluation in social interactions. Subdiagnostic ADHD was associated with more severe subdiagnostic social anxiety in adolescents, compared to those without ADHD (community-based sample: Liu et al., 2014). Furthermore, comorbid SAD and ADHD was associated with more symptoms of social anxiety and reduced social functioning, as well as with more symptoms of ADHD.

Greenberg and De Los Reyes (2022) found that adolescents with high subdiagnostic social anxiety and high subdiagnostic ADHD in a mixed clinical/community sample had significantly greater difficulties in social interactions with peers and less social skills compared to the comparison groups (which were low social anxiety/low ADHD, high social anxiety/low ADHD, and low social anxiety/high ADHD). Umutlu et al. (2022) found that adults with comorbid social phobia and ADHD in a clinical sample reported significantly lower levels of empathy compared to those without ADHD. Furthermore, Koyuncu et al. (2017) found that adults with comorbid SAD and ADHD (clinical sample) scored significantly higher on a measure of sensitivity to non-verbal cues and rejections. University students with subdiagnostic social anxiety and subdiagnostic ADHD also had a higher neuroticism personality score and a lower extraversion score compared to students without ADHD (Evren et al., 2017).

Comorbid SAD and ADHD was also shown to exacerbate ADHD symptoms. For example, studies found that (1) more symptoms of diagnostic social phobia was a significant predictor of diagnostic ADHD (Karam et al., 2015), and that (2) social phobia contributed to the persistence of ADHD (Karam et al., 2017). Comorbid diagnostic SAD and ADHD was associated with fewer years spent in education (Anker et al., 2018). Koyuncu et al. (2015) reported that the lifetime prevalence of other comorbid disorders was significantly higher in adults with both SAD and ADHD compared to adults with SAD only. The same study also showed that adults with both SAD and ADHD rated their overall functioning significantly lower than adults without ADHD. All of these studies included clinical samples. Taken together, the reviewed studies indicated a higher severity of symptoms for individuals with comorbid SAD and ADHD.

### Does the Literature on Comorbid SAD and ADHD Recognize a Subtype of SAD High on Impulsivity?

Regarding the existence of a highly impulsive subgroup of social anxiety, three of the included studies were relevant (Christian et al., 2021; Lipton et al., 2016; Michelini et al., 2015). The first two samples comprised university/college students, and the last was a community-based sample, that is, no clinical samples. Lipton et al. (2016) examined profiles of social anxiety and impulsivity in adults concerning externalizing behaviors such as alcohol use, drug use, and ADHD symptoms. Three profiles were identified in the study, and one of them comprised individuals with high social anxiety and high impulsivity. This group reported significantly higher levels of ADHD symptoms and externalizing behaviors than the other groups. Christian et al. (2021) examined the risk of psychopathology based on dimensions of perfectionism and impulsivity in adolescents and adults. The study presented four subgroups (perfectionistic/impulsive, impulsive, low both, and perfectionistic), where the combination perfectionistic/impulsive was associated with more symptoms of SAD, ADHD, and depression. Lastly, Michelini et al. (2015) reported that subdiagnostic social anxiety was positively correlated with subdiagnostic ADHD-I, but not with the hyperactivity/impulsivity subgroup.

## Discussion

The aim of this systematic review was to systematically compile empirical data on the link between SAD and ADHD in terms of the co-prevalence of the disorders in adolescents and adults, the symptom burden of comorbid SAD and ADHD, and whether the included studies recognized a highly impulsive subgroup of social anxiety. There was a wide variation in reported prevalence ratings across the 41 included studies. Surprisingly, most studies were conducted with adults, even though adolescence is known to be a particularly vulnerable period for individuals with SAD and ADHD (Gardner & Gerdes, 2015; Haller et al., 2015; McKay et al., 2023). Overall, the review indicated a broad prevalence range for either disorder in the other, perhaps partly due to how the disorders were measured (i.e., diagnostic vs. subdiagnostic, diagnostic interviews vs. symptom scales, clinical vs. general population etc.).

Comorbid SAD and ADHD was associated with greater social difficulties including more severe symptoms of social anxiety. Karam et al. (2015, 2017) found that diagnostic SAD worsened the prognosis for persons with diagnostic ADHD as it contributed to the persistence of ADHD symptoms over time. If Koyuncu et al.’s (2018) developmental hypothesis holds true, suggesting that persons with ADHD are at risk of developing SAD due to difficulties in social situations, it supports screening of SAD in patients with ADHD. Another possible explanation for this association is the link between social anxiety, attention to the self, and excessive fear of being scrutinized by others (Clark & Wells, 1995; Rapee & Heimberg, 1997), wheras ADHD often results in actual “failures” in social situations due to inattention or impulsivity (Gardner & Gerdes, 2015). Koyuncu et al. (2017) found that individuals with comorbid SAD and ADHD exhibited higher sensitivity to non-verbal cues and rejections, known as interpersonal or rejection sensitivity, compared to individuals with SAD only. Rejection sensitivity may lead to avoiding situations where persons face the risk of rejection or criticism. Others may try to minimize the risk of rejection by ensuring close relationships, which can result in more aggressive behaviors (Canu & Carlson, 2007). Future research could delve deeper into understanding the interplay between rejection sensitivity, SAD, and ADHD to gain insights into potential underlying mechanisms and to develop more targeted interventions for persons with comorbid SAD and ADHD.

Earlier studies have identified a subgroup of individuals with high impulsivity and high social anxiety, also termed *atypical social anxiety* (Kashdan et al., 2009; Mörtberg et al., 2014; Tillfors et al., 2021). Only two of the included studies mentioned a subgroup that at least partly fit this description. Lipton et al. (2016) described a group of individuals with subdiagnostic social anxiety and high impulsivity which reported relatively more ADHD symptoms and externalizing behaviors. Likewise, the perfectionistic/impulsive group in Christian et al. (2021) reported relatively more symptoms of SAD, ADHD and depression. One possible explanation for the largely missing identification of this subgroup might be a lack of association between a highly impulsive subtype of social anxiety (i.e., atypical social anxiety) and ADHD. Another, perhaps more likely explanation, is the lack of research studies focusing on subtypes of comorbid SAD and ADHD (Kashdan et al., 2009; Tillfors et al., 2021). A third explanation lies in the typical approach to studying comorbidity. The standard way of examining latent heterogeneity among individuals on constructs of interest relies on the use of normally distributed continuous (or dimensional) variables to investigate how people or objects differ quantitatively along continua of interest (Collins & Lanza, 2010; Field, 2018), via so-called *variable-centered approaches*. Nevertheless, there is growing interest in the literature to adopt *person-oriented approaches* to study qualitative differences between people, which would allow for disentangling diverse patterns of mental, biological, and/or behavioral factors operating at all levels of individual functioning. As such, person-oriented approaches are particularly useful in the study of individual development of psychopathology, as they allow for identifying significant inter-individual differences that are not automatically aligned with the “average” symptom presentation (Bergman et al., 2003). As most of the studies reviewed in this paper adopt a variable-oriented approach, it is therefore no wonder researchers have not identified the combination of high impulsivity and high social anxiety, especially as it represents a counterintuitive trait combination.

Several studies found SAD to be more common in combination with the inattentive form of ADHD rather than with the combined form of ADHD. Koyuncu et al. (2015) reasoned that individuals with the inattentive form of ADHD may appear more passive and shy compared to those with the combined form (including symptoms of hyperactivity and impulsivity), which could partially explain this pattern. Similarly, Michelini et al. (2015, p. 427) concluded that “the association between anxiety and ADH problems is driven by the association between anxiety and attention problems, but not hyperactivity/impulsivity.” This is also in line with a previously recognized subtype of ADHD and comorbid anxiety with increased attentional problems and *decreased* impulsivity (Brown, 2000; Jensen & Cantwell, 1997; Pliszka et al., 1999). So called Sluggish Cognitive Tempo (SCT) symptoms, characterized by reduced mental alertness and confusion, daydreaming etc., has also been found to be associated with less hyperactivity/impulsivity, and associated with more internalizing disorders (Schatz & Rostain, 2006). Of note, there has been a recent shift in terminology, where SCT has been replaced with Cognitive Disengagement Syndrome (Becker et al., 2023). These results regard anxiety disorders lumped together, that is, not only SAD. Moreover, studies on ADHD subtypes should be interpreted with caution, as there are uncertainties regarding their reliability, validity and stability over time (Gorlin et al., 2016; Schatz & Rostain, 2006).

Although most studies were conducted in Europe and North America and include people living in other parts of the world (US, Turkey, Brazil, Japan, Germany, Korea, Australia, Canada, France, Greece, Iceland, Netherlands, New Zealand, Norway, UK, Sweden, and Taiwan), the ethnic diversity of participants within each study was not analyzed. This can be problematic as both the prevalence of SAD and how SAD is manifested are associated with cultural factors (e.g., perceived social norms in a given cultural group; Hofmann et al., 2010). Additionally, three of the studies included culture-specific diagnoses of social anxiety, namely hikikomori (Umeda et al., 2021) and Tajin-Kyofu-sho (Kajitani et al., 2019; Kajitani et al., 2021). More research studies on cultural differences concerning SAD in general, and specifically comorbid SAD and ADHD, is therefore certainly warranted. In addition, most studies focus on the two dominant sexes (i.e., male and female). The gender perspective is relevant for the interpretation of the results (e.g., external validity) as women are more likely to be diagnosed with SAD (Asher et al., 2017), and men with ADHD (Rucklidge, 2014). Two studies were conducted with male participants only, one with incarcerated adults (Einarsson et al., 2009), and one with military servicemen (Yeom et al., 2020), and the rest of the studies included samples of both sexes. As other genders were not mentioned, however, the results can only be generalized to biological sex—a common issue with most research studies. Furthermore, we did not focus on socio-economic factors in the analysis, which might also be relevant to the generalizability of the results as such variables constitute major risk factors for the most common mental disorders, social anxiety included (McLaughlin et al., 2012). The same goes for ADHD: lower socioeconomic is associated with a higher prevalence of the diagnosis (e.g., Russell et al., 2015). At the same time, people with higher socioeconomic status are more likely to obtain prescribed ADHD medications (Simoni & Drentea, 2016), adding to the structural healthcare inequalities. Most of the included studies were cross-sectional, which prevented us from identifying any causal links between SAD and ADHD. Therefore, future studies with longitudinal designs should investigate whether one disorder constitutes a risk factor for the development of the other. Additionally, the majority of the included studies were conducted with adults despite the relevance of adolescence for this context, whereas longitudinal studies following individuals from childhood or adolescence into adulthood would yield important insights into the co-development of diagnostic and subdiagnostic ADHD and SAD.

This study has several limitations. First, a common risk when conducting systematic reviews is the possibility of missing relevant studies or including non-relevant ones, which can lead to bias in the results and negatively impact the validity of the review. By only including peer-reviewed, published articles like we have done, the results may be affected by a potential publication bias (Hesser & Andersson, 2015), that is, misleading results. For example, we have excluded so-called “gray literature.” Second, the qualitative ratings of the 41 studies were remarkably low, so our conclusions should be seen as preliminary. Nevertheless, although we used a qualitative assessment tool for quantitative empirical studies, that assessment tool is not adapted for studies using cross-sectional designs, which was the nature of the majority of the included studies. Interestingly, when we also excluded the study aspect “confounders” in addition to the three study aspects we excluded from start (since the included studies did not include interventions), the overall picture of the quality ratings changed from being remarkably low to being of more moderate quality. Hence, this indicates that our conclusions are more reliable than the quality ratings initially indicated. A third limitation is the use of different terms for subdiagnostic and diagnostic SAD and ADHD in the included studies (e.g., social phobia). A fourth limitation was that only some of the included studies measured anxiety disorders other than SAD, and when they did, other anxiety disorders were not necessarily controlled for. A fifth limitation was the fact that some of the included studies were based on the DSM-IV and others on the DMS-5 diagnostic criteria of SAD and/or ADHD, potentially impacting the reliability and comparability of findings. Future reviews on the relationship between SAD and ADHD should consider setting the time limit at 2013 instead of 2000, that is, when the DSM-5 was published (APA, 2013).

Despite these limitations, the current study has a number of strengths. First, we have formulated focused questions based on previous research. Second, we have conducted systematic searches and documented the process carefully to increase replicability. Third, even if we included only peer-reviewed published articles, our literature searches covered several large databases as well as backward searches of the included articles’ reference lists and forward searches via their citations, which partly reduces the risk for overlooking relevant studies. Fourth, our quality assessment employed established criteria. Lastly, we have integrated the results and highlighted recommendations for both future research and the clinic.

The current systematic literature review showed wide-ranging prevalence rates of SAD in adolescents and adults with ADHD, and vice versa. We identified several important knowledge gaps, particularly concerning these links in adolescence, prompting more research studies with this age group. Comorbid SAD and ADHD was associated with more severe symptoms and higher levels of impairment compared to having one of the disorders. Thus, for earlier detection of potential comorbidity, individuals with either diagnosis should be screened for the other. Clinicians should also make note of subdiagnostic symptoms of both SAD and ADHD—perhaps especially the inattentive subgroup of ADHD—as even subdiagnostic symptoms can worsen the prognosis in terms of outcomes. More focus on subdiagnostic links between high impulsivity and social anxiety, as well as how they overlap with clinical diagnoses of ADHD and SAD would be desirable. A focus on well-designed and well-conducted studies on the link between SAD and ADHD would enable the investigation of mechanisms at work, helping us to potentially prevent some of the negative outcomes related to this counterintuitive comorbidity. Finally, by elucidating what is unique to SAD compared to anxiety in general, researchers can provide a more nuanced understanding of the relationship between SAD and ADHD, ultimately informing more targeted interventions and treatments for adolescents and adults experiencing these comorbidities.

## Supplemental Material

sj-docx-1-jad-10.1177_10870547241247448 – Supplemental material for The Relationship Between Social Anxiety Disorder and ADHD in Adolescents and Adults: A Systematic Review Supplemental material, sj-docx-1-jad-10.1177_10870547241247448 for The Relationship Between Social Anxiety Disorder and ADHD in Adolescents and Adults: A Systematic Review by Siri Jakobsson Støre, Nejra Van Zalk, Wilma Granander Schwartz, Victoria Nilsson and Maria Tillfors in Journal of Attention Disorders
