# Supplementary material for: The Relationship Between Social Anxiety Disorder and ADHD in Adolescents and Adults: A Systematic Review
Source: J Atten Disord. 2024 Apr 23;28(9):1299–319. doi: 10.1177/10870547241247448 (PMC11168018; doi:10.1177/10870547241247448)
Supplement: sj-docx-1-jad-10.1177_10870547241247448 – Supplemental material for The Relationship Between Social Anxiety Disorder and ADHD in Adolescents and Adults: A Systematic Review [file sj-docx-1-jad-10.1177_10870547241247448.docx]

*Table S1.* *Quality assessment of the included studies (excluding confounding variables).*

| Authors (year) | Selection bias | Study design | Method för data collection | Attrition | Overall |
| --- | --- | --- | --- | --- | --- |
| Anker et al. (2018) | M | W | S | N/A | **M** |
| Becker et al. (2015) | W | W | S | N/A | W |
| Christian et al. (2021) | W | W | S | N/A | W |
| Coskun et al. (2020) | W | W | M | N/A | W |
| Edel et al. (2010) | W | W | S | N/A | W |
| Einarsson et al. (2009) | W | W | S | N/A | W |
| Evren et al. (2017) | S | W | S | N/A | **M** |
| Fredrick et al. (2020) | W | W | S | N/A | W |
| Gorlin et al. (2016) | W | W | S | N/A | W |
| Greenberg & De Los Reyes (2022) | W | M | S | N/A | **M** |
| Kajitani et al. (2019) | W | W | M | N/A | W |
| Kajitani et al. (2021) | W | W | M | N/A | W |
| Karam et al. (2015) | M | M | S | M | **S** |
| Karam et al. (2017) | M | M | S | N/A | **S** |
| Koyuncu et al. (2017) | W | W | S | N/A | W |
| Koyuncu et al. (2019) | W | W | S | N/A | W |
| Koyuncu et al. (2015) | W | W | M | N/A | W |
| Lipton et al. (2016) | W | W | S | N/A | W |
| Liu et al. (2014) | S | W | S | N/A | **M** |
| Marmorstein (2006) | S | W | S | N/A | **M** |
| Marmorstein (2007) | S | W | S | N/A | **M** |
| Michelini et al. (2015) | M | W | M | N/A | **M** |
| Moore et al. (2016) | M | W | M | N/A | **M** |
| Mörtberg et al. (2012) | W | M | S | N/A | **M** |
| O'Rourke et al. (2020) | W | M | S | N/A | **M** |
| Park et al. (2011) | S | W | S | N/A | **M** |
| Pehlivanidis et al. (2020) | W | M | M | N/A | **M** |
| Peyre et al. (2022) | W | M | S | N/A | **M** |
| Pine et al. (2000) | S | W | S | N/A | **M** |
| Rucklidge et al. (2016) | W | M | S | N/A | **M** |
| Safren et al. (2001) | W | M | M | N/A | **M** |
| Secnik et al. (2005) | W | M | W | N/A | W |
| Thoma et al. (2020) | W | M | M | N/A | **M** |
| Umeda et al. (2021) | W | W | S | N/A | W |
| Umutlu et al. (2022) | W | M | M | N/A | **M** |
| Van Ameringen et al. (2011) | W | W | M | N/A | W |
| Vogel et al. (2018) | M | W | S | N/A | **M** |
| Wilens et al. (2005) | W | M | S | N/A | **M** |
| Yeom et al. (2020) | S | W | M | N/A | **M** |
| Yoldas et al. (2019) | W | M | M | N/A | **M** |
| Yüce et al. (2013) | W | W | S | N/A | W |

*Note. S = Strong, M = Moderate, W = Weak, N/A = Not Applicable. Overall ratings in bold letters are those that changed (24 out of 41 studies) when the confounder variable was excluded.*
